# Regulatory T Cells As Supporters of Psychoimmune Resilience: Toward Immunotherapy of Major Depressive Disorder

**DOI:** 10.3389/fneur.2018.00167

**Published:** 2018-03-20

**Authors:** Pierre Ellul, Encarnita Mariotti-Ferrandiz, Marion Leboyer, David Klatzmann

**Affiliations:** ^1^Sorbonne Université, Assistance Publique - Hôpitaux de Paris (AP-HP), Robert Debré Hospital, Department of Child and Adolescent Psychiatry, Paris, France; ^2^Sorbonne Université, INSERM, Immunology-Immunopathology-Immunotherapy (i3), AP-HP, Hôpital Pitié-Salpêtrière, Biotherapy (CIC-BTi) and Inflammation-Immunopathology-Biotherapy Department (i2B), Paris, France; ^3^Team 15, INSERM U955 Institut Mondor de Recherche Biomédicale (IMRB), Créteil, France; ^4^Faculté de Médecine, Université Paris-Est Créteil Val de Marne (UPEC), DHU PePSY, Pôle de Psychiatrie et d’addictologie, Hôpitaux Universitaires Mondor, Assistance Publique - Hôpitaux de Paris (AP-HP), Créteil, France; ^5^Fondation FondaMental, Créteil, France

**Keywords:** regulatory T cell, major depressive disorders, inflammation, immunotherapy, low-dose interleukin-2

## Abstract

There is growing evidence that inflammation plays a role in major depressive disorder (MDD). As the main role of regulatory T cells (Tregs) is to control inflammation, this might denote a Treg insufficiency in MDD. However, neither a qualitative nor a quantitative defect of Tregs has been ascertained and no causality direction between inflammation and depression has been established. Here, after reviewing the evidence supporting a relation between Treg insufficiency and MDD, we conclude that a novel therapeutic approach based on Treg stimulation could be valuable in at least the subset of patients with inflammatory MDD. Low-dose interleukin-2 appears to be a good candidate as it is not only a safe stimulator of Tregs in humans but also an inhibitor of pro-inflammatory Th17 lymphocytes. Here, we discuss that a thorough immune investigation as well as immunotherapy will be heuristic for deciphering the pathophysiology of MDD.

## Introduction

### On Inflammation and Major Depressive Disorder (MDD)

Major depressive disorder is a disabling psychiatric disorder that afflicts more than 10% of the adult population in the USA and is associated with a global social cost of 66.5 million disability-adjusted life years (1). Currently ranked as the fourth cause of disability and premature death in the world, MDD is expected to be the second leading contributor to overall disease burden by 2030 (2). Approximately one in six individuals will suffer from MDD once in their lifetime (3). The first episode of MDD is often the onset of a chronic relapsing and remitting illness that leads to an increased premature mortality and morbidity primarily attributable to suicide and cardiovascular-related disorders (4). Despite well-conducted antidepressant treatment, approximately one-third of all patients with depression fail to respond to conventional antidepressant therapies (5).

The role of inflammation in depression has recently been reviewed in depth (6). The observation of an immune inflammatory response to stress, together with the link between stress and depression, establishes a first indirect link between inflammation and depression (7–9). The risk of depression is high in many disorders with an inflammatory component, including cardiovascular diseases, diabetes, metabolic syndrome, infections, and autoimmune disorders such as rheumatoid arthritis or psoriasis (10, 11). Reinforcing the link between inflammation and depression, genetic studies have shown that mutation in inflammatory-related genes (such as CRP or IL-6) increased the risk on MDD onset (12–14).

Meta-analysis of clinical studies in MDD has highlighted increased blood levels of various pro-inflammatory cytokines and their soluble receptors, such as interleukin-6 (IL-6), tumor necrosis factor-alpha (TNF-alpha), IL-1β, soluble IL-2 receptor, and C-reactive protein (CRP), in MDD compared to healthy controls (15–17). It has been estimated that 47% of MDD patients have a CRP level of >3.0 mg/L and 29% a CRP level of >5.0 mg/L, leading to the concept that MDD is mediated by inflammation (18). Altogether, although there is no direct evidence of causality, depression and inflammation appear to be interconnected, possibly fueling and feeding off each other. Thus, when inflammation and depression cooccur, treating both of them may enhance recovery from both disorders, reduce the risk of recurrence, and effect improvement in patients with a resistant depression (19).

Further support in favor of the role of inflammation in depression comes from the clinical use of pro- or anti-inflammatory treatment. The treatment of normal individuals with drugs that have an inflammatory effect, such as interferon-gamma, is associated with symptoms of depression (20, 21). By contrast, depression has been improved by anti-inflammatory treatments, particularly in subgroups of MDD patients with low-grade inflammation (22, 23). A trial with add-on infliximab (an inhibitor of the inflammatory TNF-alpha) has revealed an increased clinical response compared with antidepressants alone in patients with treatment-resistant depression, but only in those with a CRP of >5 mg/L (22). Several other studies are ongoing in order to evaluate the antidepressant effect of anti-inflammatory drugs (24–26).

### On Inflammation and Regulatory T Cells (Tregs)

The main role of Tregs is to control inflammation and immune tolerance, the two being interrelated (27). Tregs act by inhibiting pro-inflammatory cellular responses and by secreting anti-inflammatory cytokines (28). The experimental ablation of Tregs in an otherwise healthy animal leads to severe inflammation (29). Likewise, any inflammatory or autoimmune disease might denote, in essence, the inability of Tregs to control inflammation, thus a Treg insufficiency (30). It could be due to a decreased number or function of Tregs or, conversely, to an increased number or function of effector T cells (Teffs), as well as to the microenvironment of the immune response, which would affect Treg efficiency. Tregs are thus key targets for the treatment of many autoimmune and inflammatory diseases, in particular by their activation/induction through the administration of low doses of IL-2 (ld-IL-2) (30). Interestingly, the proof-of-concept trial of ld-IL-2 in an autoimmune disease showed not only that it safely activates Tregs but also that it has an overall anti-inflammatory effect (31). Indeed, the changes in the overall transcriptomic profile of peripheral blood mononuclear cell induced by ld-IL-2 clearly revealed an anti-inflammatory pattern (31). In addition, ld-IL-2 was also shown to improve chronic graft-versus-host disease, which is considered as an alloantigen-induced chronic inflammation, after hematopoietic stem cell transplantation (32).

In this review, we discuss how Treg insufficiency may contribute to the pathogenesis of MDD and likewise how Treg stimulation with ld-IL-2 can be envisioned as a treatment for MDD patients with low-grade inflammation and possibly beyond.

## Physiology and Regulation of Tregs

The *Forkhead box P3* (FoxP3) transcription factor and the alpha chain of the IL-2 receptor (CD25) are the markers of Tregs (33). We recently reviewed their role and regulation by IL-2 (30). We here summarize the aspects of their physiology that pertain to their role in depression.

The major roles of Tregs are to control inflammation and prevent autoimmune diseases (33). Indeed, the efficient depletion of Tregs in healthy individuals at any time in life induces a rapid inflammatory response that leads to multiorgan autoimmunity (29). This revealed that, in healthy individuals, there are Teffs ready to attack normal tissues, but they are kept under control by Tregs. This led to the concept that there is a Treg/Teff balance in health, which is dysregulated in autoimmune and inflammatory disorders. In humans, many but not all autoimmune diseases have been associated with a qualitative or a quantitative defect of Tregs, such as in type 1 diabetes, multiple sclerosis, rheumatoid arthritis, and systemic lupus erythematous (34–36). However, even for diseases without a Treg deficiency, the failure to control inflammation denotes Treg insufficiency (30).

Since their discovery, decades of studies have revealed that Tregs are in fact heterogeneous in terms of origin, phenotype, and function. Ontogeny distinguishes Tregs that differentiate during T cell development in the thymus (tTregs) from peripheral Tregs (pTregs) that develop from naïve CD4^+^ cells upon TCR/CD28 costimulation in the presence of cytokines such as TGF-β and IL-2 (37). tTreg cells are considered to be relatively homogeneous and stable in comparison with pTregs (34, 37, 38). History of activation distinguishes naïve Tregs from activated/memory Tregs, the latter being essentially self-antigen-specific (39) and enriched in deep lymphoid organs (40–42). Tissue localization then distinguishes (i) circulating Tregs, which are found in secondary lymphoid organs and fluids, from (ii) tissue-resident Tregs, which are resident in non-lymphoid tissues such as gut, fat, and skin (33). All these characteristics are closely linked to the role of these cells in specific settings. Resident Tregs have been described as having specific functions—often trophic—linked to their environment. For instance, in the muscle, Tregs can contribute to muscle repair by the production of amphiregulin (43). Tregs in the brain can also have trophic functions as they promote oligodendrocyte differentiation and re-myelination (44).

In humans, Tregs are mainly discriminated based on the combination of FOXP3^hi^CD25^hi^CD127^lo^ markers, with CD45RO^+^ activated/memory or CD45RA^+^ naïve phenotypes (45).

IL-2 is the key cytokine that regulates the development, homeostasis, and function of Tregs. Mice that do not produce IL-2 or the high-affinity IL-2 receptor die of severe lymphoproliferative-driven autoimmunity (46), a phenomenon later explained by a failure in Treg (47). By contrast, the development of pro-inflammatory Th17 and T-follicular helper cells is opposed by IL-2 (48, 49). Patients with autoimmune diseases such as type 1 diabetes (50), rheumatoid arthritis (51), and systemic lupus erythematosus (52), compared with healthy individuals, have a genetically determined low IL-2 production, which reduces their Treg fitness. The role of IL-2, and likewise of Tregs, in autoimmune diseases was recently further highlighted by the meta-analysis of a shared genetic architecture across 10 pediatric autoimmune diseases which revealed a central role of the IL-2 pathway (53).

Although Tregs control inflammation, inflammation controls Tregs (30). Tregs tend to lose their functional capacity and become unstable in highly inflammatory environment (54, 55). On the other hand, Tregs suppress inflammation by multiple mechanisms, including reducing costimulation to activate Teffs, consuming IL-2, and secreting immunosuppressive cytokines such as IL-10 (56). They also stimulate dendritic cells to produce the anti-inflammatory regulatory enzyme indolamine 2,3 dioxygenase (IDO), which in turn activates Tregs and suppresses inflammation in part by tryptophan consumption (57, 58). These anti-inflammatory effects of Tregs are confirmed by their capacity to improve various models of inflammatory diseases in mice, such as in atherosclerosis, acute lung injury, muscular dystrophy, and beryllium-induced granulomatous inflammation (35). In these models, ld-IL-2 stimulates Tregs and improves the conditions (30). These results highlight the broad therapeutic potential of IL-2 for tipping the balance of Treg/Teff cells toward Treg cells. Some Tregs may experience a phenotypic plasticity, notably related to the instability of FoxP3 expression leading to a possible Th1/Th17 polarization (33, 36, 37). This could notably be an issue for Treg cell-based therapy in high inflammatory conditions. However, (i) thymic-derived Tregs that constitutively express CD25 are the main target of ld-IL-2 and are not prone to such instability; (ii) when present, the inflammation associated with MDD is a mild systemic inflammation rather than a focal high inflammation like in some autoimmune diseases, and (iii) the activation of the STAT5 pathway by IL-2R signaling actually contributes to stabilize FOXP3 expression and likewise the Treg-suppressive phenotype. These observations are in line with the fact that no Treg instability has been described in the clinical trials investigating ld-IL-2 in over 20 different clinical settings.

## Tregs and the Pathophysiology of MDD

### Immunopathophysiology of MDD

It is known that stress, a major risk factor of MDD, is associated with the activation of the hypothalamic–pituitary–adrenal (HPA) axis, leading to the release of catecholamine (6). Catecholamine seems to act on the pathophysiology of MDD through damage-associated molecular patterns, which eventually activate the NLRP3 inflammasome (6, 59), a pro-inflammatory multiprotein complex activated by pathogenic microorganisms and by sterile stressors (e.g., ATP, oxidative stress). The inflammasome is responsible for secretion of the inflammatory IL-1β (60), which in turn could drive the inflammatory response associated with MDD. Pro-inflammatory cytokines can mediate brain inflammation either directly by crossing the blood–brain barrier or indirectly (6, 59). IL-1β inhibits the enzyme tetrahydrobiopterin, which is essential for the synthesis of dopamine, therefore decreasing the availability of this neurotransmitter (6, 61). Possibly in response to the increase in pro-inflammatory cytokines in MDD, the activation of IDO has been observed (59, 62). This leads to a decrease of the neurotransmitter serotonin (5-HT) and an increase in N-methyl-d-aspartate signaling (metabotropic glutamate receptor). The coexistence of increased IDO, the anti-inflammatory role of which is in part mediated by Tregs, with an inflammatory state is paradoxical and could suggest a defect in Tregs in MDD mediated by inflammation. Altogether, several lines of evidence suggest that inflammatory processes play an important role in MDD, but it should be recognized that these lines of evidence are quite indirect.

### Tregs in MDD

Studies in rodents indicate that Tregs may play a protective role against depressive-like behavior. In animal models of depression, using the chronic unpredictable mild-stress paradigm, a decrease in Tregs has been shown to be significantly associated with the onset of depressive-like behavior (63). *In vivo*, Treg depletion prior to exposure of mice to stress led to a higher rate of depressive-like behavior and pro-inflammatory profile compared to untreated mice (64). In a rat model of postpartum depression, Tregs were negatively associated with pro-inflammatory cytokines. In addition, the treatment of mice with the antidepressant fluoxetine is associated with an increased percentage of Tregs (65). Finally, it has been shown that in response to acute stress, as well as during repeated stress, the activation of the HPA axis promotes immune cell trafficking (59).

Importantly, the transfer of T cells from mice exposed to chronic social defeat, but which do not develop depressive-like behavior, to naïve mice led to an antidepressant behavioral phenotype (6). This is one rare direct line of evidence linking Tregs and behavior.

In humans, adolescents at a high risk for mood disorder exhibit a decreased number of Tregs that is negatively correlated with their inflammatory state (66). Similarly, several studies have found a decrease in Tregs in MDD patients (67–69) that is also associated with a pro-inflammatory phenotype in those patients (68).

Thus, both human and animal studies show an association between an increased risk of MDD and a decreased number of Tregs associated with an increased inflammation. Since depressed patients resistant to antidepressant treatment are mostly those with an inflammatory profile, the effect of treatment on Tregs has been studied. In MDD patients with decreased Tregs at baseline compared with non-Treg-related MDD, Tregs are increased during effective antidepressant treatment, whatever antidepressant is prescribed (70). This suggests that Tregs may be a predictor of treatment (70, 71).

However, it should be kept in mind that the associations described above do not prove causality. Social factors and habits, as well as the disease itself, may influence diet, hygiene, and exposure to infectious agents, all of which can affect immune homeostasis and/or influence the microbiota and likewise the inflammatory context.

## Hypothesis on Mechanisms Linking Treg Insufficiency and MDD

### Gut Microbiota, Tregs, and MDD

There is now robust evidence that the microbiota influences the immune system and *vice versa*. More specifically, there are close interactions between the gut microbiota and Tregs (72–74): (i) while there are numerous Tregs in the intestinal mucosa at the steady state, germ-free mice have a reduced number of such Tregs; (ii) some specific strains of bacteria have been associated with more inflammatory or more regulatory-tuned intestinal milieu; (iii) the transfer of gut microbiota from mice with autoimmune diseases is sufficient to transmit the disease to germ-free mice; and (iv) by contrast, the transfer of defined Clostridium strains was recently shown to induce Tregs and improve colitis and allergic diarrhea in mice (75, 76). With this in mind, there are different lines of evidence suggesting a “ménage à trois” between the gut microbiota, Tregs, and MDD. MDD is known to be associated with an increased intestinal permeability or “leaky gut,” for which there is no clear explanation (62). Since Tregs are major sensors of immune tolerance in the gut, one can hypothesize a causal (forward or reverse) link between the deregulation of Tregs, “leaky gut,” and MDD. In this regard, low levels of vitamin D, which affect microbiota composition and impair Treg activation, correlate with MDD onset (77–79), and, reciprocally, vitamin D supplementation decreases depressive symptoms and oxidative stress markers in MDD patients (80). Similarly, low fatty acid levels have also been associated with MDD onset (81, 82). A meta-analysis of 13 studies including 1,233 MDD patients revealed that antidepressant plus fatty acid supplementation was more effective than antidepressant alone (83). Interestingly, this effect is especially seen in patients with an inflammatory profile (23). Early psychic trauma has been shown to alter gut microbiota (84). One explanation for this gut–brain crosstalk is that it is sustained by the immune system (85). It is worth noting that fecal transplantation of microbiota from patients with MDD to germ-free mice results in depressive-like behaviors (84), which is direct evidence of a causal link between MDD and microbiota.

### Adipose Tissue, Tregs, and MDD

Metabolic syndrome is defined by lipid disturbance, insulin resistance, and abdominal obesity and has been extensively associated with the onset of MDD, especially in young patients (86). Obesity comprises an inflammatory disorder in which fat Tregs seem to play an important role (87). Inflammation related to obesity is in part related to the onset of MDD (10). An elevated body mass index and waist circumference are correlated with an increased risk of MDD (88, 89). Obese patients with MDD exhibit a poorer response to standard antidepressant treatments (90). There are different lines of evidence suggesting another “ménage à trois,” between obesity, fat Tregs, and MDD. High leptin levels lead to obesity, and the administration of leptin has also been shown to increase depressive-like behaviors in mice (91). In humans, several studies have found an association between high leptin levels and the onset of MDD in obese patients (92–94). Altogether, as leptin is a potent antagonist of fat Tregs, the above results suggest a causal link between high leptin level, Treg insufficiency, and MDD. Conversely, PPAR gamma is a major regulator of adipogenesis (95, 96). It also contributes to Treg activation and development, especially in Tregs from adipose tissues (97). Interestingly, it has been shown that PPAR gamma agonists such as pioglitazone have the ability to decrease depressive symptoms in MDD (98, 99).

### Interleukin-2 (IL-2) and MDD

Interleukin-2 is the major cytokine needed for the activation of Tregs. sIL-2R (sCD25), the soluble form of IL-2Ra (CD25), is increased in patients with MDD (17, 100–102). The biological activity of sIL-2R is not known and its affinity for IL-2 is low. Nevertheless, sCD25 could decrease the bioavailability of IL-2, which in turn would impact on Treg survival and expansion.

In conclusion, many of the commonly reported abnormalities found in MDD could contribute or be linked to a defect in Treg activation.

## Psychoimmune Pharmacotherapy: Modulation of Immunity to Regulate Mood

One-third of all patients with depression fail to respond to conventional antidepressant therapies (5). Forty-seven percent of them exhibit a CRP of >3 mg/L (22) and thus have an inflammatory condition characterizing Treg insufficiency, which may not only be involved in the onset of MDD but could also be associated with treatment resistance. Treg stimulation could thus be used as an adjunct treatment of MDD—particularly in resistant-depressed patients—which could reverse the inflammation and possibly neurotransmitter modification observed in MDD patients.

**Figure 1:**
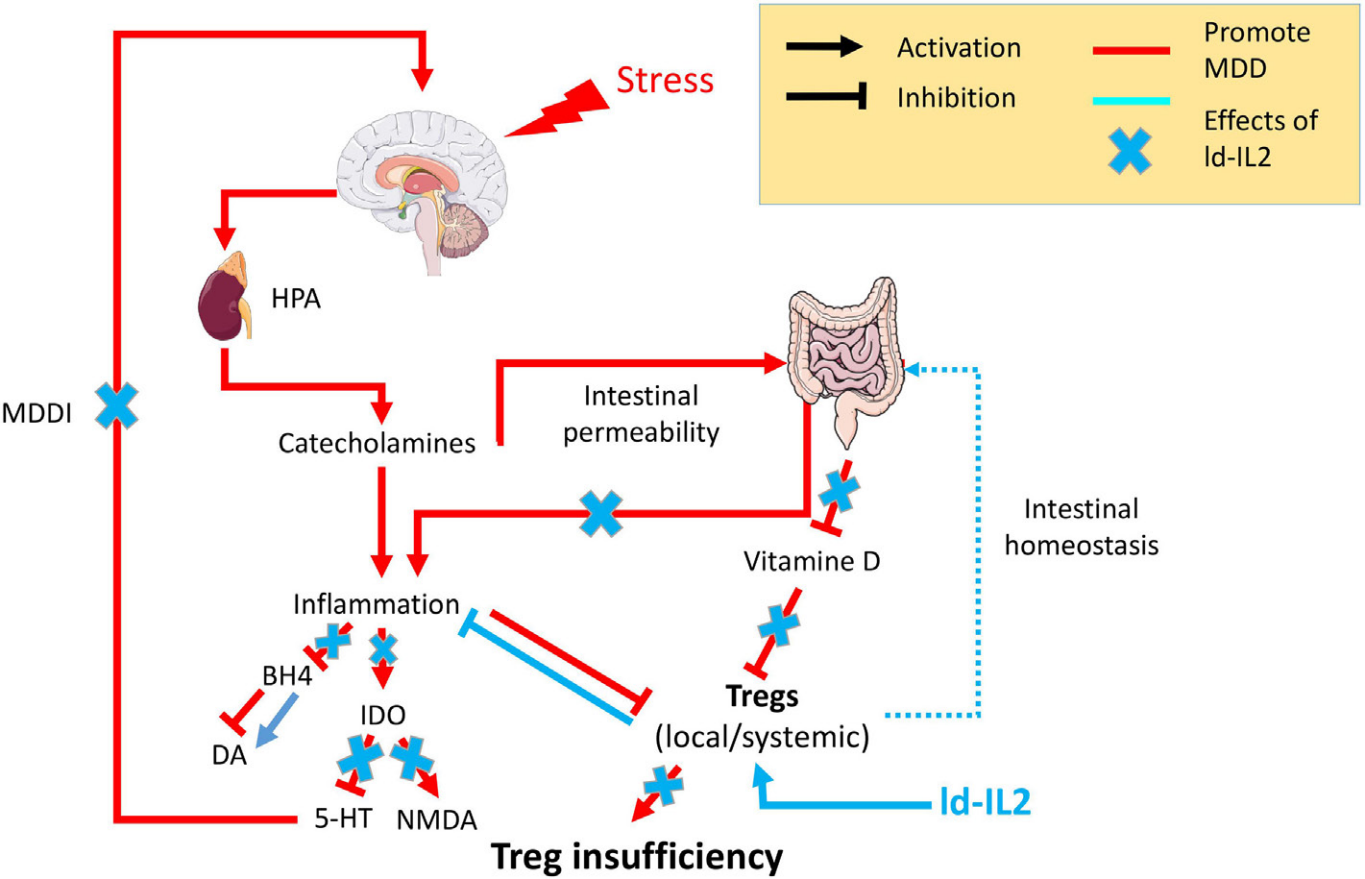
Psychological stress factors are associated with the activation of the hypothalamic–pituitary–adrenal axis (HPA) leading to the release of catecholamine, which in turn are responsible for low-grade inflammation. This inflammation will be responsible for (i) an increased intestinal permeability, the so-called “leaky gut,” which in turn fuels inflammatory processes, (ii) inhibition of the enzyme tetrahydrobiopterin (BH4), which decreases the availability of the neurotransmitter dopamine (Da), (iii) chronic activation of the anti-inflammatory regulatory enzyme indolamine 2,3 dioxygenase (IDO) leading to a decrease of the neurotransmitter serotonin (5-HT) and an increase in N-methyl-d-aspartate (NMDA) signaling. The cooccurrence of inflammation with other factors [vitamin D or regulatory T lymphocyte (Treg) deficiencies] will prevent Tregs’ control of the inflammatory state. The resulting chronic modification in neurotransmitters will be responsible for the onset of major depressive disorder mediated by inflammation (MDDI). The use of low-dose interleukin-2 (ld-IL-2), by stimulating Tregs, will control the inflammatory environment, reestablish both neurotransmitters and intestinal homeostasis, and in so doing prevent or reverse MDDI symptoms.

Interleukin-2, the Treg master regulator, has pleiotropic functions. It can induce the development of thymic and pTregs, but also maintains their functional competence and stability (30). IL-2 is also able to support the proliferation of Teffs such as CD4^+^ and CD8^+^. Nevertheless, Tregs have a greater avidity for IL-2 due to their constitutive expression of high-affinity IL-2R, leading to a 10- to 20-fold lower activation threshold for IL-2R signaling than Teffs and a >100-fold increase in sensitivity to gene activation downstream of IL-2R (103). This raises the possibility of achieving specificity for Treg stimulation by using ld-IL-2 (30). In 2006, ld-IL-2 was used for the first time with the aim of stimulating Tregs in 10 patients with hepatitis C virus-induced vasculitis. ld-IL-2 was well tolerated and expanded and activated Tregs without activating Teffs, which led to significant clinical improvements (31). Since then, ld-IL-2 has been evaluated in many other diseases. For example, in chronic graft-versus-host disease, ld-IL-2 improved the manifestation of the disease at multiple sites in the majority of glucocorticoid-resistant patients (32). In alopecia areata patients, ld-IL-2 induced the recruitment of Treg around hair follicles and a dramatic hair regrowth in a majority of patients (104). In SLE, multiple studies have shown a dramatic decrease of disease activity in most of the patients included (105) (Humrich team). Phase-IIb double-blind placebo-controlled trials are now underway, and their results should help better define the efficacy of the treatment. All these studies have confirmed the excellent safety profile of ld-IL-2 and have sometimes indicated remarkable clinical improvements related to Treg expansion. Several additional clinical trials evaluating ld-IL-2 in multiple autoimmune and inflammatory diseases are ongoing, including TRANSREG (NCT01988506), which includes patients suffering from 1 of 11 selected autoimmune diseases. This study currently shows that ld-IL-2 treatment is well tolerated even in the long term (106). Altogether, the safety of ld-IL-2, its effects on boosting Tregs and inhibiting Teff cells, and Treg insufficiency strongly support its clinical investigation in MDD (Figure 1).

## Conclusion

Regulatory T cells appear to link many biological abnormalities found in MDD, shedding light on how stress, inflammation, and neurobiological modifications can be linked to its pathophysiology. Tregs can also explain the associations between MDD and many inflammatory diseases related to Treg insufficiency, explaining the high rate of depression in these populations. As such, Tregs could be envisioned as a biomarker of MDD as they can be measured accurately and reproducibly and as a potential therapeutic target in MDD patients. Likewise, the use of ld-IL-2 as an adjunct to antidepressant treatment holds great promise in MDD and more largely in the field of psychiatric treatment. The correlative data linking Tregs and MDD together with the safety, tolerability, and efficacy profile of ld-IL-2 should prompt clinical evaluation of this novel therapeutic approach. Incidentally, a clinical benefit of ld-IL-2 treatment in MDD will not only help alleviate the disease burden but also provide evidence of a direct contribution of the immune system (and Tregs) to psychiatric disorders.

## Author Contributions

PE researched data for the article and wrote the first version of the article. All authors, discussed, wrote, edited, and reviewed the manuscript before submission.

## Conflict of Interest Statement

PE, EM-F, and ML declare no competing interests. DK is an inventor of a patent application claiming low-dose IL-2 for therapy of autoimmune diseases, owned by his academic institution and licensed to ILTOO Pharma, which he advises and in which he holds shares.
